# Laws governing access to sexual health services and information: contents, protections, and restrictions

**DOI:** 10.1080/26410397.2024.2336770

**Published:** 2024-04-22

**Authors:** Laura Ferguson, Sarah Emoto, Sofia Gruskin

**Affiliations:** a Director of Research, Institute on Inequalities in Global Health, University of Southern California, Los Angeles, CA, USA; b Program Specialist, Institute on Inequalities in Global Health, University of Southern California, Los Angeles, CA, USA; c Director, Institute on Inequalities in Global Health, University of Southern California, Los Angeles, CA, USA

**Keywords:** sexual health, sexual health services, law, sexual rights, non-discrimination

## Abstract

Access to sexual health services and information is critical to achieving the highest attainable standard of sexual health, and enabling legal environments are key to advancing progress in this area. In determining overall alignment with human rights standards to respect, protect, and fulfil sexual health-related rights without discrimination, there are many aspects of laws, including their specificity and content, which impact which sexual health services and information are availed, which are restricted, and for whom. To understand the nature of existing legal provisions surrounding access to sexual health services and information, we analysed the content of 40 laws in English, French, and Spanish from 18 countries for the specific sexual health services and information to which access is ensured or prohibited, and the non-discrimination provisions within these laws. Overall, there was wide variation across countries in the types of laws covering these services and the types and number of services and information ensured. Some countries covered different services through multiple laws, and most of the laws dedicated specifically to sexual health addressed only a narrow aspect of sexual health and covered a small range of services. The protected characteristics in non-discrimination provisions and the specificity of these provisions with regard to sexual health services also varied. Findings may inform national legal and policy dialogues around sexual health to identify opportunities for positive change, as well as to guide further investigation to understand the relationship between such legal provisions, the implementation of these laws within countries, and relevant sexual health outcomes.

## Plain language summary

Access to sexual health services and information is important to being able to have good sexual health. Laws are relevant because what they include and how specific they are affects what types of sexual health services people can access, what types of services are illegal, and whether or not all people can access services equally. We reviewed 40 laws in English, French, and Spanish from 18 countries to understand how many and which sexual health services and information countries ensure in their laws, which sexual health services are illegal, and which people are protected from discrimination in accessing these services. We found that countries use many different types of laws to ensure access to sexual health services or information, and most countries do not cover the same types or number of sexual health services. There are also differences in which people are specifically protected from discrimination in the laws we reviewed. These findings are important because they may help countries identify ways that access to sexual health services and information could be improved so as to improve people’s sexual health. They may also guide future research.

## Résumé

L’accès aux services et à l’information en matière de santé sexuelle est capital pour parvenir au niveau de santé sexuelle le plus élevé possible, et des environnements juridiques favorables sont essentiels pour progresser dans ce domaine. Lorsqu’on détermine l’alignement global sur les normes des droits de l’homme pour respecter, protéger et réaliser les droits relatifs à la santé sexuelle sans discrimination, de nombreux aspects des lois, notamment leur spécificité et contenu, influent sur les informations et services de santé sexuelle qui sont utilisés, lesquels sont restreints et pour qui. Afin de comprendre la nature des dispositions juridiques existantes concernant l’accès à l’information et aux services de santé sexuelle, nous avons analysé le contenu de 40 lois en anglais, en français et en espagnol dans 18 pays pour l’information et les services de santé sexuelle spécifiques auxquels l’accès est garanti ou interdit, et les dispositions de non-discrimination contenues dans ces lois. Dans l’ensemble, il existait de grandes variations entre pays dans les types de lois couvrant ces services ainsi que dans les types et le nombre de services et d’informations proposés. Certains pays englobaient différents services par le biais de plusieurs lois, et la plupart des lois consacrées spécifiquement à la santé sexuelle n’abordaient qu’un aspect restreint de la santé sexuelle et couvrait une petite gamme de services. Les caractéristiques protégées dans les dispositions de non-discrimination et la spécificité de ces dispositions par rapport aux services des santé sexuelle variaient également. Ces observations peuvent éclairer les dialogues juridiques et politiques nationaux autour de la santé sexuelle pour identifier les possibilités de changement positif, ainsi que guider des recherches plus approfondies en vue de comprendre la relation entre ces dispositions juridiques, la mise en œuvre de ces lois dans les pays et les résultats pertinents en matière de santé sexuelle.

## Resumen

El acceso a información y servicios de salud sexual es fundamental para alcanzar el más alto nivel de salud sexual, y los entornos legislativos favorables son clave para lograr avances en esta área. Para determinar la alineación general con las normas de derechos humanos de respetar, proteger y cumplir los derechos relacionados con la salud sexual sin discriminación, hay muchos aspectos de las leyes, tales como su especificidad y contenido, que influyen en qué información y cuáles servicios de salud sexual son aprovechados, cuáles son restringidos y para quién. Para entender la naturaleza de las disposiciones legislativas vigentes relativas al acceso a la información y los servicios de salud sexual, analizamos el contenido de 40 leyes en inglés, francés y español de 18 países para saber a qué información y servicios específicos de salud sexual el acceso es garantizado o prohibido, y las disposiciones de no discriminación establecidas en estas leyes. En general, hubo gran variación entre países en los tipos de leyes relativas a estos servicios y los tipos y la cantidad de servicios e información garantizados. Algunos países abarcaban diferentes servicios por medio de varias leyes, y la mayoría de las leyes dedicadas específicamente a la salud sexual abordaban solo un aspecto limitado de la salud sexual y una gama pequeña de servicios. También variaron las características protegidas en las disposiciones de no discriminación y la especificidad de estas disposiciones con respecto a los servicios de salud. Los hallazgos podrían informar los diálogos nacionales sobre leyes y políticas relativas a la salud sexual para identificar oportunidades de realizar cambios positivos, así como para guiar más investigaciones a fin de entender la relación entre tales disposiciones legislativas, la aplicación de estas leyes en los países y los resultados de salud sexual relevantes.

## Introduction

Access to sexual health services and information is critical to achieving the highest attainable standard of sexual health, which the World Health Organization (WHO) defines as a state of physical, emotional, mental, and social well-being in relation to sexuality, and not merely the absence of disease.^1^

The United Nations (UN) Sustainable Development Goals (SDGs) affirm a global political commitment to increasing access to sexual health services and information through three key targets. SDG 3.7 aims to ensure universal access to sexual and reproductive health services, including family planning, information, and services. SDG 5.6 calls for ensuring universal access to sexual and reproductive health and reproductive rights with indicator 5.6.2 tracking the number of countries with laws and regulations guaranteeing full and equal access to sexual and reproductive health care, information, and education for women and men ages 15 and older.^2,3^ Alongside the SDGs’ overarching commitment to non-discrimination through the pledge to “leave no one behind”, target 10.3 is to ensure equal opportunity and reduce inequalities, including through the elimination of discriminatory laws.^2^ In addition to the SDGs, other global strategies, such as the Global Strategy for Women’s, Children’s and Adolescents’ Health and the Global AIDS Strategy 2021–2026, recognise enabling legal environments as key to improving access to sexual health services and information.^4,5^

The legal foundation for states’ obligations with regard to sexual health is found in international human rights law. Binding international human rights treaties specify states’ obligations regarding the right to health, understood to include sexual health. Article 12 of the International Covenant on Economic, Social and Cultural Rights (ICESCR) recognises the right to the highest attainable standard of health without discrimination.^6^ The Committee on Economic, Social and Cultural Rights (CESCR), in its interpretation of Article 12 in General Comment No. 22, further expounds on countries’ legal obligations to respect, protect, and fulfil rights related to sexual and reproductive health, including to guarantee universal and equitable access to affordable, acceptable and quality sexual and reproductive health services, and to repeal laws that create barriers to access.^7^ The Committee on the Elimination of All Forms of Discrimination against Women in its interpretation of the obligation to eliminate discrimination against women in access to healthcare states that “States parties should ensure, without prejudice or discrimination, the right to sexual health information, education and services for all women and girls”.^8^ In its interpretation of the child’s right to health, the Committee on the Rights of the Child notes that “States should ensure that adolescents are not deprived of any sexual and reproductive health information or services due to providers’ conscientious objection”.^9^

The extent to which countries incorporate international human rights standards into their national legislation impacts individuals’ abilities to access sexual health services and information. Laws provide the framework for how sexual health policies, programmes, and services are implemented, including which sexual health services and information are ensured, which are restricted, and for whom.^10^ In addition to laws specific to the provision of health services, other relevant criminal, civil or administrative laws may facilitate or create barriers to access.^10^ For example, punitive laws that criminalise HIV transmission or non-disclosure may prevent individuals from seeking and accessing critical services, including HIV testing, prevention, or treatment.^11^ Such laws that create barriers to HIV services have cascading effects including the violation of individual rights, increased inequalities for marginalised groups, and broader consequences for population health and economic development. Conversely, enabling laws that ensure access without discrimination can facilitate uptake of services and are key for promoting sexual health and for effective HIV prevention and response.^11^ Laws which include non-discrimination provisions can mitigate inequalities in who is able to access such services and information. Non-discrimination provisions in laws related to sexual health are critical not only for ensuring equal access to sexual health services but also to ensure protection against practices that violate individual rights, such as involuntary or coerced sterilisation.^12^ As such, there are many aspects of laws to consider in determining overall alignment with human rights standards to respect, protect, and fulfil the right to sexual health without discrimination.

Previous studies have made critical advancements in research on laws related to sexual health, such as laws related to HIV services,^13^ laws that criminalise sex work,^14^ and non-discrimination laws with respect to sexual orientation.^15^

Work on abortion laws has highlighted an increasingly apparent need to examine more closely not just the existence of laws, but also the specificity of their provisions. Studies focused on the language of abortion laws illustrate how and why the specificity in the law matters for those seeking to access abortion, as specifications regarding gestational age limits, mandatory waiting periods, or administrative requirements, such as authorisation from multiple physicians, can result in restricted access, even where abortion is considered legal, and even where laws are considered liberal based on broad legal grounds for abortion.^16–20^

Other studies have examined how the specificity of laws, or lack thereof, relates to adolescents’ abilities to access sexual health services. For example, whether an age of consent for services is specified, whether adolescents are explicitly mentioned in laws relating to sexual health services, and if there are inconsistencies between the minimum age for consent to different services and for consent to sexual activity all have implications for adolescents’ access to services.^21–24^

A global analysis of national constitutions assessed whether and to what extent they respect, protect, and fulfil the right to sexual and reproductive health, to what extent they are inclusive and non-discriminatory, and whether they acknowledge linkages between the right to sexual and reproductive health and other human rights.^25^ The findings usefully draw attention to how the specificity in provisions may impact an individual’s ability to access services, even when constitutions contain specific provisions for services.

While this existing literature has highlighted the value of deeper content analysis of laws related to specific sexual health services or populations and useful approaches for performing such analyses, there remains a gap in understanding the specificity and content in laws governing sexual health services and information across different types of law.

### The WHO Global Policy Survey (GPS)

In order to track countries’ progress in the adoption of WHO recommendations across sexual, reproductive, maternal, newborn, child, and adolescent health (SRMNCAH) laws, policies, strategies, and guidelines, WHO developed the SRMNCAH GPS.^26,27^ While previous iterations of the survey were conducted for maternal, child and adolescent health, the latest version, administered in 2018, was the first to include sexual and reproductive health.^26^ Additionally, the 2018 GPS was designed not only to assess if countries have certain SRMNCAH laws and policies in place, but also whether they contain provisions in alignment with the commitments in the SDGs and the Global Strategy for Women’s, Children’s and Adolescents’ Health.^26^ Importantly, the 2018 GPS, which was administered online, requested that countries upload legal and policy source documents to support their survey responses.

### Research aims

The overarching research question for this analysis was *What is the nature of existing legal provisions to ensure access to sexual health services and information without discrimination?* Given the significance of the role of law in promoting or restricting access to sexual health services and information, this research aims to bridge a gap in the current literature using the findings and documents from the 2018 GPS to analyse and understand the legal content, protections, and restrictions related to access to sexual health services and information. This provides important insights into how countries’ laws cover, or fail to cover, relevant rights and obligations for sexual health services and information.

## Methods

We carried out a content analysis of the laws that were uploaded by countries to support their responses to the sexual health questions in the 2018 GPS. Even as some laws will have changed since this time, the 2018 GPS constitutes the most current global publicly-available dataset at time of writing. We systematically extracted information from each law on:

- provisions to ensure access to sexual health services and information, including social and legal services for sexual health in these laws;
- provisions which prohibited sexual health services and information in these laws; and
- provisions for non-discrimination contained within these laws.

### Structure and content of the sexual health sub-section of the GPS

Sexual health is a sub-section within the reproductive health module of the 2018 GPS. The first question asked if countries have national policies or laws on sexual health services and information.^28^ If countries responded “no” or “unknown” to this question, they were instructed to skip the entire sexual health sub-section. If countries responded “yes”, they were asked the following additional questions covering a range of issues relating to sexual health services and information:

- Do the laws or policies on sexual health information and services have provisions for non-discrimination for the following groups?
  - Age, sex, sexual orientation, gender identity, disability, race/ethnicity, marital status, HIV status, involvement in sex work, others.
- Are there laws or policies on any of the following?
  - Decriminalising male sex workers, decriminalising female sex workers, regulating sex work through regular medical checkups.
- Are there policies/laws prohibiting harmful practices related to sexual health?
- Do any policies/laws exist that prohibit the following?
  - Female genital mutilation (FGM) and virginity testing.
- Are there policies or laws establishing mandatory Comprehensive Sexuality Education (CSE) as part of the regular educational curriculum?
- Do policies or laws exist on standard curricula for the training of health providers in sexuality counselling?
- Do policies or laws exist on a strategy to provide sexuality counselling (as defined by WHO) through public services?

Countries were then asked to upload documents to support their answers for the eight preceding questions.

In the “cross-cutting” module of the survey, another question asked “Does the country have a dedicated law on sexual health?”^28^ and documents to substantiate this were also requested.

### Inclusion criteria for laws

Laws were identified from WHO’s master document repository catalogue from the 2018 SRMNCAH GPS. We grouped inclusion criteria in two ways. For the first grouping, the criteria were: the country reported having a national law or policy on sexual health services and information in the reproductive health module and uploaded at least one law to substantiate this answer. For the second grouping, the criteria were: the country answered “no” to having a national law or policy on sexual health services and information in the reproductive health module but “yes” in the cross-cutting module and uploaded at least one law to substantiate this answer. This approach was chosen because, while we anticipated capturing any laws related to sexual health services and information based on the first set of criteria, we realised that some countries that had not reported having a law on sexual health services or information in the reproductive health module did report having a sexual health law in the cross-cutting module; thus, adding the second set of criteria expanded our dataset to include additional countries.

Identified laws were included if they were in English, French, or Spanish (based on the language skills of the research team).

### Data analysis

Each law that met the inclusion criteria was reviewed and systematic data extraction was performed with the following categories of information: types of sexual health service(s) and information to which the law ensures access, sexual health services and information expressly prohibited by law, non-discrimination provisions, and the supporting textual evidence for each category. LF performed data extraction for laws in French and Spanish and SE for laws in English. LF and SE performed the data analysis.

The following definitions were used during this process.

*Sexual health services:* prevention of and treatment for HIV, sexually transmitted infections (STIs), reproductive tract infections (RTIs), and their adverse outcomes (such as cancer and infertility), contraception, abortion (and post-abortion care), sexual dysfunction, the sequelae of sexual violence, FGM, and sexual and reproductive cancers. This also includes social and legal services related to sexual health, such as identification and referral for victims of sexual violence and FGM. This list of services was derived from definitions of sexual health services identified in WHO documents.^10,29^

*Sexual health information:* provision of information relating to prevention of and treatment for HIV, STIs, RTIs, and their adverse outcomes (such as cancer and infertility); contraception, abortion (and post-abortion care), sexual dysfunction, the sequelae of sexual violence, FGM, and sexual and reproductive cancers. This includes CSE, as well as information about relevant social and legal services.

*Non-discrimination provision:* a provision within a law that proscribes discrimination against individuals based on certain characteristics.

With the goal of only focusing on laws that covered sexual health services and information, the laws were then categorised into four groups:

1. Specific laws containing text relevant to sexual health services and information, covering laws that are specifically dedicated to sexual health services, information, or non-discrimination in the provision of sexual health services or information.
2. Non-specific laws containing text relevant to sexual health services and information, including laws containing general non-discrimination provisions, but that are not themselves dedicated to this topic (e.g. penal codes, constitutions, general health laws, child rights acts, and adolescent laws);
3. Laws containing text relevant to social or legal services for sexual health (non-medical services); and
4. Non-relevant laws containing text with no relevant information for this study (i.e. laws that were uploaded to substantiate answers to survey questions not being analysed here).

We analysed the content of the laws in categories 1–3 to identify provisions which ensure access to sexual health services and information or provisions which prohibit access to sexual health services and information. Even as some laws are explicit in their guarantee of some services, for the purposes of this analysis, we use “ensure access” to also include legal provisions that govern ensuring access to services even as they may not contain a legal guarantee. For the laws in category 1, we also assessed whether the law contained explicit mention of the WHO definition of sexual health. This allowed us to assess the extent to which WHO’s normative guidance is explicitly adopted in national laws specifically dedicated to sexual health. For the laws in categories 1–3, we assessed the non-discrimination provisions, including alignment of provisions with those contained in international human rights documents. Codes for this analysis were derived deductively from the WHO definition of sexual health services above and the categories of discrimination as elaborated in international human rights documents. We inductively added additional codes for relevant services or non-discrimination characteristics that appeared in the laws reviewed.

## Results

In this section, we present an overview of the dataset that we used for our analysis, followed by our findings on the content of laws relating to the use of the WHO definition for sexual health, provisions that ensure access to sexual health services and information, provisions that prohibit sexual health services, and provisions for non-discrimination, including their alignment with international human rights standards.

### Number and distribution of laws

Overall, 111 (72%) out of 155 countries that completed the survey reported, in either place in the survey, that they have a national law or policy on sexual health services and information. Twenty countries uploaded a total of 57 laws in English, French, or Spanish to substantiate their responses.

Given the focus of this study, laws were reviewed and categorised based on their content (Table 1). For the analysis of the provisions in these laws, only relevant laws were considered, totalling 40 documents from 18 countries. Five laws, two from India, and one each from Argentina, Mexico, and Venezuela fell into more than one category.

**Table 1.** Categorisation of laws uploaded, per country and WHO Region

Country and WHO region	1: Specific laws containing relevant text to sexual health services and information	2: Non-specific laws containing relevant text to sexual health services and information	3: Laws containing relevant text to social or legal services for sexual health
***African Region***	***0***	***3***	***1***
Uganda	0	1	1
Zimbabwe	0	2	0
***Americas Region***	***6***	***13***	***4***
Argentina	0	3	1
Barbados	0	0	1
Bolivia	0	4	0
Costa Rica	1	2	0
Cuba	1	0	0
Mexico	1	2	1
Nicaragua	2	2	0
Venezuela	1	0	1
***Eastern Mediterranean Region***	***0***	***1***	***0***
Djibouti	0	1	0
***European Region***	***1***	***4***	***0***
Estonia	0	2	0
Luxembourg	0	2	0
Malta	1	0	0
***South East Asia Region***	***5***	***2***	***3***
India	5	0	3
Nepal	0	2	0
***Western Pacific Region***	***1***	***1***	***0***
Brunei Darussalam	0	1	0
Philippines	1	0	0
**Total number of laws**	**13**	**24**	**8**

### Content of relevant laws: sexual health services and information

This section provides an overview of the relevant content within the laws that fell into categories 1–3 outlined above. First, findings on usage of the WHO definition of sexual health within laws are outlined. Then, information is presented on the types of laws that ensure access to sexual health services or information and the relevant content of these laws, with specific attention to those services where access is ensured, those prohibited by law, and restrictions to access for minors. Finally, the content of social and legal services relating to sexual health is provided.

#### Usage of the WHO definition of sexual health

Of the 13 laws specific to sexual health services or information, only one law from India explicitly uses the WHO definition of sexual health.^30^

#### Laws ensuring access to sexual health services or information

Twenty-nine laws from 14 different countries included provisions that ensured access to sexual health services or information in some way. These laws varied widely. Some covered sexual and reproductive health broadly, others addressed more narrow issues of sexual health, such as gender-based violence, sexual offences, sexuality education, discrimination against people of diverse sexual orientations and gender identities, and certain aspects of reproduction. For the non-specific laws that ensured access to sexual health services or information, these included laws relating to children and adolescents, general health laws, constitutions, an infectious disease act, and a law supporting access for people with disabilities. In total, the laws reviewed ensured access to 18 different types of sexual health services or types of information. Table 2 summarises these findings.

**Table 2:** . Laws ensuring access to sexual health services/information

Sexual health service and/or Information	Number of laws that ensured access to this service/information	Countries whose laws ensured access to this service/information
General medical services and information^a^	14	Argentina, Bolivia, Costa Rica, Djibouti, India, Mexico, Nepal, Nicaragua, Zimbabwe
STI/HIV treatment	8	Bolivia, Costa Rica, Djibouti, India, Mexico, Nicaragua, Philippines, Zimbabwe
Comprehensive sexuality education^b^	8	Bolivia, Cuba, Mexico, Nicaragua, Philippines
STI/HIV information/prevention	7	Bolivia, Djibouti, India, Mexico, Philippines, Nicaragua
Sexual violence medical care	7	Bolivia, India, Mexico, Nicaragua, Venezuela
Family planning	6	Bolivia, Djibouti, Mexico, Nepal, Nicaragua, Philippines
Sexual health medical services	3	Bolivia, Philippines
Sexual health counselling	4	Bolivia, Mexico, Philippines
Fertility	3	Malta, Nicaragua, Philippines
Contraception	3	Bolivia, Mexico, Philippines
Sexual violence prevention/education	3	Mexico, Nicaragua, Philippines
Sexual and reproductive cancers	3	Djibouti, Nicaragua, Philippines
HIV counselling	2	Bolivia, Brunei Darussalam
Sexual dysfunction treatment	2	Nicaragua, Philippines
Sexual and reproductive rights information	2	Bolivia
Abortion	2	Bolivia, Luxembourg
RTIs	1	Philippines
Mental health aspect of reproductive health	1	Philippines

^a^ These laws ensure access to general medical services to the population. These services could include sexual health services and information.

^b^ In Cuba and Nicaragua, the laws referenced “comprehensive sexuality education” while in Mexico the language of “comprehensive sex education” was used. In the Philippines, the law referenced “reproductive health and sexuality education.”

The service most frequently ensured by law was access to general medical services and information, which was identified in 14 laws from nine countries. For the purposes of this analysis sexual health services were assumed to be covered as they fall within the ambit of general medical services, however, eight out of the nine countries that addressed general medical services did not provide the explicit confirmation that sexual health services might be included. Only one country, Zimbabwe, had two laws that specified reproductive health services as a subset of general medical care, however, these laws did not detail the specific services included within reproductive health or address sexual health more broadly.^31,32^

STI/HIV treatment was the service that was next frequently ensured by law: it was covered in eight laws from eight countries. Most laws ensuring access to this service specifically mentioned treatment of STIs or HIV, however Zimbabwe’s Public Health Act ensured a right for people living with a chronic illness to access services for the illness, which is presumed to include HIV, even as the law did not specify.^32^ Of the eight countries that ensure access to STI/HIV treatment, five countries (Bolivia, Djibouti, India, Mexico, Philippines, Nicaragua) also ensure access to STI/HIV information/prevention.

Of the 14 countries which ensured access to sexual health services or information, 10 included four or fewer services; of these, five countries only addressed one service, and there was no pattern as to which service. The four countries which covered the most comprehensive services were the Philippines,^13^ Bolivia,^12^ Nicaragua,^10^ and Mexico.^9^ The Philippines uploaded one specific law, its Responsible Parenthood and Reproductive Health Act, covering 13 different services or types of information, spanning many different areas of sexual health: sexuality education, STI/HIV prevention and treatment, fertility, family planning, contraception (but not emergency contraception), sexual health medical services, reproductive health and sexuality counselling, sexual violence prevention and education, treatment of sexual dysfunction, RTIs, and reproductive cancers, and mental health (articulated in the law as “mental health aspect of reproductive care”). Bolivia uploaded three non-specific laws that guaranteed services, and most of its 12 services were covered in its child and adolescent code. Nicaragua uploaded four laws, two of which were specific to sexual health services – a sexual and reproductive health law and a law on the protection of families with multiple pregnancies or births – and two non-specific laws, a general health law and a law on child and adolescent rights. Most of the services were covered in its sexual and reproductive health law; however, general medical services, sexual and reproductive cancers, and sexual violence prevention/education were covered across its other three laws. Mexico uploaded three laws, one specific to gender violence, and two non-specific laws, a general health law and law on child and adolescent rights. Similar to Bolivia, most of the sexual health services and information were covered in the child and adolescent law.

#### Abortion: a service both ensured and prohibited in laws

Abortion was the only sexual health service within the definition noted above that was identified as a service both ensured and prohibited in the laws reviewed.

Two laws from two countries ensured access to abortion. Bolivia’s child and adolescent code ensures abortion services, including information, services, follow-up, and psychological services, but only for girls and adolescents who are victims of sexual violence.^33^ Luxembourg’s law on the adoption of the Convention on the Rights of the Child (CRC) specifies that the right to life in the CRC must not interfere with national legal provisions on access to sexuality information, prevention of clandestine abortions, and regulation of abortion.^34^ In contrast, Argentina’s law on the approval of the CRC^35^ states that the CRC applies, beginning at conception. Bolivia and Costa Rica also state that life begins at conception in their child and adolescent codes.^33,36^ India uploaded its Medical Termination of Pregnancy Act, which specifies that abortion is legal on broad grounds, however it does not explicitly ensure access to this service.^37^

Five countries uploaded laws banning all or specific types of abortion. Malta,^38^ Nicaragua,^39^ and the Philippines^40^ prohibited abortion in laws specific to sexual health services and information, whereas Argentina^41^ and Costa Rica^42^ uploaded their penal codes. The Philippines’ law, where it proscribes abortion, also proscribes “management of abortion complications”, which conflicts with a different clause that obligates the government to ensure women needing care for post-abortion complications are treated and counselled in a humane, non-judgmental, and compassionate manner in accordance with law and medical ethics.^40^

#### Other sexual health services prohibited by law

In addition to abortion, the Philippines’ law specifically prohibits emergency contraceptive pills.^40^

Other reproductive services that fell outside the agreed upon definition of sexual health services and information but were prohibited by law were also identified. One of India’s laws, for example, prohibits sex selection of an embryo (including in the case of IVF) and also prohibits the identification of the sex of an unborn child.^43^ Malta’s law prohibits specific embryo services such as fertilising more than five eggs, embryo experimentation, selection of sex, cloning, and the alteration of a germline.^38^

#### Restrictions for minors in accessing sexual health services and information

Two laws specified restrictions for minors accessing sexual health services and information. The Philippines requires written parental consent for minors to access family planning services, except in cases where the minor is already a parent or has had a miscarriage, but does not provide an exception if the minor is married.^40^ The Philippines’ law does not explicitly define minors. India’s Medical Termination of Pregnancy Act requires written parental consent for minors under 18 to access abortion, and does not specify any exceptions.^37^

#### Social and legal support services for sexual health

In addition to the medical sexual health services and information in the laws, social and legal services relating to sexual health were also covered in some laws. Seven countries uploaded nine laws containing information on social and legal support relevant to sexual health, all of which addressed services related to harmful sexual practices. India uploaded three laws specifying social or legal services: a trafficking law which outlined legal and social services for victims of trafficking such as providing victims female welfare workers, the right to a hearing, and protective homes;^44^ an act on the protection of children from sexual offences which ensures access to legal assistance for child victims;^45^ and an act prohibiting child marriages, which ensures that child marriages shall be legally voidable at the option of the person who was a child at time of marriage.^46^ Barbados’ sexual offences act gives any victim of a sexual offence the right to a legal hearing.^47^ Mexico’s gender violence law includes information on legal services for female victims of gender violence as well as educational programmes against gender violence for perpetrators.^48^ Nepal’s constitution states that women shall not be subjected to sexual violence, and that victims shall have the right to compensation in accordance with the law. It also gives the right to compensation from the perpetrator for victims of child marriage and child sexual abuse.^49^ Uganda’s law on FGM states that perpetrators of FGM must pay for the medical services victims require.^50^ Venezuela’s gender violence law similarly states that perpetrators of gender violence must pay for the medical services that victims require and that women have access to social and legal services for gender-based violence.^51^ Argentina’s law on the protection of the rights of children and adolescents ensures access to social or protective services, such as scholarships for education, medical treatment, or financial support, for children whose rights are threatened or violated.

### Content of relevant laws: non-discrimination provisions

#### Non-discrimination provisions

Twenty-five laws from a total of 14 countries contained some form of a non-discrimination provision, covering 27 characteristics. Countries varied widely on which and how many characteristics they included in their non-discrimination provisions: Bolivia (19), Zimbabwe (17), Mexico (16), Nicaragua (15), Costa Rica (14), Nepal (13), Uganda (13), Estonia (10), Argentina (10), India (8), Philippines (6), Cuba (3), Malta (3), and Venezuela (2). The three most common characteristics addressed in the non-discrimination provisions were sex, religious beliefs, and race/ethnicity/colour. Involvement in sex work was the only characteristic from the list in the GPS question which was not included in any non-discrimination provisions. Table 3 details this information.

**Table 3.** Characteristics found in non-discrimination provisions

Characteristic	Number of laws containing non-discrimination provision for this characteristic	Countries whose laws contain non-discrimination provisions for this characteristic
Sex	19	Argentina, Bolivia, Costa Rica, Estonia, India, Malta, Mexico, Nepal, Nicaragua, Zimbabwe
Religious beliefs	16	Argentina, Bolivia, Costa Rica, Estonia, India, Mexico, Nepal, Nicaragua, Philippines, Uganda, Venezuela, Zimbabwe
Race/ethnicity/colour	15	Argentina, Bolivia, Costa Rica, Estonia, India, Mexico, Nepal, Nicaragua, Uganda, Venezuela, Zimbabwe
Age	13	Argentina, Bolivia, Costa Rica, Cuba, Estonia, Mexico, Nicaragua, Philippines, Uganda, Zimbabwe
Origin/nationality	11	Argentina, Bolivia, Costa Rica, Estonia, India, Mexico, Nepal, Nicaragua, Uganda, Zimbabwe
Disability	11	Argentina, Bolivia, Costa Rica, Estonia, India, Mexico, Nicaragua, Uganda, Zimbabwe
Political or philosophical beliefs or opinions	11	Argentina, Bolivia, Estonia, Mexico, Nepal, Nicaragua, Uganda, Zimbabwe
Sexual orientation	11	Bolivia, Costa Rica, Estonia, India, Malta, Mexico, Nicaragua
Economic status/property	11	Argentina, Bolivia, Costa Rica, Estonia, Mexico, Nepal, Nicaragua, Uganda Zimbabwe
Social status	11	Bolivia, Costa Rica, Estonia, Mexico, Nicaragua, Uganda Zimbabwe
Gender identity	10	Bolivia, Costa Rica, Cuba, India, Malta, Mexico, Nicaragua, Philippines, Uganda, Zimbabwe
Language	10	Bolivia, Costa Rica, Estonia, Mexico, Nicaragua, Uganda, Zimbabwe
Culture	10	Argentina, Bolivia, Costa Rica, Nepal, Nicaragua, Uganda, Zimbabwe
Marital status	6	Bolivia, Costa Rica, Mexico, Nepal, Philippines, Zimbabwe
Health status	5	Argentina, Bolivia, Mexico, Nepal, Nicaragua
Intellect/education	3	Bolivia, Nicaragua
Profession/occupation	3	Bolivia, Costa Rica, Philippines
Pregnancy	3	Bolivia, Nepal, Zimbabwe
Born in/out of wedlock	3	Mexico, Nepal, Zimbabwe
Tribe	2	Nepal, Zimbabwe
Caste	2	India, Nepal
Class	1	Zimbabwe
Environmental factors	1	Bolivia
HIV status	1	Cuba
Personal circumstances	1	Philippines
Migrant status	1	Mexico
Family status	1	Uganda

Not every country that ensured access to a sexual health service or information or social and legal services relevant to sexual health uploaded a law with a non-discrimination clause. Barbados, Brunei Darussalam, and Djibouti uploaded laws that ensured access to services without including any form of non-discrimination provisions.

#### Content of non-discrimination provisions

The way countries included non-discrimination provisions in the laws varied from country to country and from law to law. Most non-discrimination provisions were covered in non-specific laws.

The country with the most comprehensive non-discrimination provisions was Bolivia. Bolivia’s four laws included non-discrimination provisions covering 19 out of the 27 total characteristics identified in the laws reviewed. One of their laws – a law against racism and all forms of discrimination,^52^ included a comprehensive non-discrimination provision as well as a provision specific to racial discrimination, while their general law for people with disabilities^53^ made reference to the above-mentioned law and included more detailed non-discrimination provisions based on disability. This included, for example, a provision that people with disabilities, especially women, have a right to decide freely and responsibly on matters relating to their sexuality and sexual and reproductive health free from coercion, discrimination, or violence. The country with the second most comprehensive provisions was Zimbabwe, which covered 17 characteristics in one law, its constitution.

Few laws included non-discrimination provisions specific to sexual health services and information.

Malta’s Embryo Protection Act ensures access to fertility services for all prospective parents, regardless of sexual orientation or gender.^38^

Nicaragua’s sexual and reproductive health law specifically mentions the State’s responsibility to eliminate discrimination on the basis of gender, sex, ethnicity, culture, sexual orientation, religious beliefs, political beliefs, philosophical beliefs, educational level, ability, and socioeconomic status, that might impede autonomous decision-making and equality between women and men. Furthermore, the law explicitly ensures the provision of services based on the principles of universality, equity, comprehensiveness, continuity of care, quality, complementarity, solidarity, cultural responsiveness, non-discrimination, and participation.^39^

The Philippines’ law explicitly prohibits refusing quality reproductive and sexual health services on the basis of a person’s marital status, gender, age, religious convictions, personal circumstances, or nature of work. While the Philippines’ law does not include a non-discrimination provision for persons with disabilities, the law does contain a clause on sexual and reproductive health programmes for people with disabilities, which describes obligations for municipalities to eliminate barriers for services for people with disabilities, including addressing physical access, transportation, and proximity issues; increasing access to sexual and reproductive health materials in braille, large print, simple language, sign language, and pictures; providing continuing education for health care providers on the rights of people with disabilities; and raising public awareness on the sexual and reproductive health needs and rights of persons with disabilities.

Venezuela’s gender-based violence law mentioned the non-discrimination provisions specifically applicable for ensuring access to all mechanisms to help realise the rights recognised in the law: nationality, ethnic origin, religion or any other personal, legal or social condition or circumstance.^51^

Lastly, Mexico’s gender violence law mentioned the non-discrimination provisions specifically applicable to implementation of the law – language, age, social status, sexual preference, or any other condition – and ensures access to free interpreters and public defenders with knowledge of their language and culture for indigenous women.^48^

#### Consistency with key human rights non-discrimination provisions

The prohibited grounds for discrimination as they appear in international human rights treaties have been understood to include a range of characteristics, including race, colour, sex, language, religion, political or other opinion, national, ethnic or social origin, property, birth, disability, or other status.^6,54,55^ While “other status” is understood to include a non-exhaustive list of characteristics, the CESCR and Committee on the Rights of the Child have elaborated additional specific characteristics for which discrimination is prohibited, such as marital and family status, sexual orientation, gender identity, place of residence, economic and social situation, and health status, including HIV status and mental health.^56,57^

Countries prohibit discrimination based on “other status” in different ways, with some countries naming additional characteristics (as included in the table) and others including language such as “any other condition”^58^ or “on any similar other grounds”.^49^ Thus, we did not systematically analyse the inclusion of other prohibited forms of discrimination.

All of the countries in our sample have ratified the CRC, and all but two countries have ratified both the International Covenant on Civil and Political Rights (ICCPR) and the ICESCR (Brunei Darussalam and Cuba have not ratified either). To analyse how aligned the non-discrimination provisions of these countries were with international human rights standards, the non-discrimination provisions identified in the laws uploaded were compared to the above list of characteristics. Mexico, Bolivia, Nicaragua, and Zimbabwe were most aligned with non-discrimination provisions derived from international human rights standards. Additionally, several countries referenced the international human rights treaties that they have ratified, which may, to some extent, be interpreted to imply adoption of the non-discrimination provisions contained therein. Argentina,^35^ Estonia,^59^ Luxembourg,^34^ Nicaragua,^60^ and Uganda^58^ all reference the CRC, and India^30^ references its ratification of the ICCPR and ICESCR.

### Potential conflict in laws impacting access to sexual health services and information

While identifying the types of discrimination explicitly prohibited in the laws, other factors affecting discrimination and/or potential conflict in laws relevant to sexual health were identified. Argentina, Costa Rica, and Nicaragua uploaded laws that ensure access to general medical services^36,61,62^; however they also uploaded laws that prohibit abortion.^39,41,42^ Three countries, Costa Rica,^42^ India,^44^ and Luxembourg,^63^ explicitly criminalised sex work in the laws reviewed. Argentina,^41^ Brunei Darussalam,^64^ and Zimbabwe^32^ uploaded laws which criminalise transmission of HIV. Mexico^65^ and the Philippines^40^ allow for conscientious objection in the provision of health services. Costa Rica’s penal code contained discriminatory language surrounding homosexuality which is contradictory to the non-discrimination provision for sexual orientation in its law addressing discrimination against sexually diverse individuals.^42,66^ Estonia’s Family Law Act prohibits same-sex marriage, despite its Chancellor of Justice Act ensuring a right to recourse for discrimination based on sexual orientation.^59,67^ Each of these laws, and the legal conflicts they create, has implications for limiting access to sexual health services or information, even as some do not expressly prohibit a service.

## Discussion

The findings from this analysis highlight several important implications in terms of guiding future work both at the country level and at a broader global level to strengthen laws surrounding sexual health services and information. A key strength of this analysis is that it looks across a range of sexual health services and information, using definitions provided by WHO documents, finding that, overall, the laws reviewed ensured access to very few specific sexual health services and information.

While all of the countries in this analysis answered positively either to having a law on sexual health services and information or a dedicated sexual health law, there was substantial variation with regard to the types of laws uploaded, which and how many services and types of information were included in the laws, and the level of specificity provided with regard to what is encompassed within these services and information. The number of non-specific laws was almost twice the number of laws specific to sexual health. This, coupled with the finding that the most frequently ensured service was general medical services, without explicit confirmation this includes sexual health, underscores the lack of specificity in laws addressing sexual health services and information.

Overall, the laws reviewed in this analysis demonstrate a range of different approaches to ensuring access to sexual health services in law, both in terms of looking across countries, given the variation in services covered and types of laws uploaded, as well as within countries, as different services were often covered through multiple laws, and most of the laws specific to sexual health addressed only a narrow aspect of sexual health and covered a small range of services. A dedicated sexual health law which covers sexual health services and information comprehensively may facilitate improved implementation in ensuring access to the services specified, whereas having multiple laws covering narrow aspects of sexual health may have implications for inconsistencies in implementation, allocation of resources, and translation into policy and other national guidance. For example, previous studies on age of consent laws have highlighted how a multiplicity of laws addressing age of consent to sexual intercourse, to marriage, and to accessing sexual health services results in barriers to access due to health care provider confusion about contradictory obligations for providing services, maintaining confidentiality, and mandatory reporting requirements.^68,69^ Among the four countries covering the most number of services, Nicaragua and the Philippines covered a wide range of services in a single law specific to sexual health, however Nicaragua also covered additional services in non-specific laws. The other two countries, Bolivia and Mexico, covered most of their services in non-specific laws. Future research might assess implementation in countries which cover similar services in order to compare specific and non-specific laws, as well as services ensured in a single law versus across multiple laws, in order to further understand the strengths or weaknesses of the different approaches.

The specificity and consistency of content within non-discrimination provisions also varied from country to country. Most non-discrimination provisions were in non-specific laws and were not specific to the provision of sexual health services or information. While general provisions for non-discrimination may sufficiently apply to sexual health services, it may also be important to consider how laws related to sexual health services may need to specifically address certain characteristics in order for services and information to be accessible in practice. Few of the laws reviewed included a non-discrimination provision on the basis of health status and only one specifically included HIV status. Depending on the country context, specificity for non-discrimination in the provision of sexual health services may be critical to ensuring access for certain marginalised populations.

In terms of consistency with international standards, only India explicitly makes reference to the WHO definition for sexual health in its law; including the WHO definition within laws may provide a stronger legal grounding for ensuring access to the types of relevant services and information necessary to achieve sexual health. The non-discrimination provisions in the laws from Mexico, Bolivia, Nicaragua, and Zimbabwe are most closely aligned with the non-discrimination provisions that are predominant in international human rights documents; it would be interesting to know the history of how these laws were developed to better understand if these processes might be replicable elsewhere. In addition to international standards, it may also be useful to examine how national laws governing sexual health services and information are consistent with regional level standards. Previous work has underscored the value of examining regional documents in relation to sexual health, both to use as a measure of comparison for national laws, as well as to understand how the specificity in regional legal frameworks and the strength of accountability mechanisms have implications for the interpretation and operationalisation into national legislation.^21,70,71^ Where potential conflicts in laws exist at the national level, or between those laws and regional or international standards, it will be important to explore avenues for accountability.

In the laws reviewed, the only social or legal services specifically ensured addressed sexual violence. Other relevant social and legal services are also integral to sexual health and in some cases may be critical to accessing medical services, for example, assistance for transgender persons in securing legal identification to reflect their gender.^72^ The lack of identified provisions ensuring access to social or legal services beyond sexual violence may point toward a need for further research into other types of non-medical services related to sexual health and how effective legal reform might address these.

### Limitations of data

Relatively few relevant laws were available from the 2018 GPS in English, French, or Spanish, which limited the overall findings as well as regional and language comparisons. It would be useful to replicate this content analysis on a wider data set of laws relating to sexual health services and information to further analyse patterns in legal content across additional countries and languages. This analysis was based on documents provided, on the unchecked assumption that these are the most recent applicable laws in each country; it is possible that more recent laws exist. Additionally, other relevant laws may exist that were not uploaded to the identified sub-sections of the GPS. The next round of GPS should be available soon and might constitute an expanded database for this type of analysis. Once the next iteration of the GPS is published, future research could include temporal analysis and examine changes in countries’ legal environments in the context of progressive realisation and/or retrogression in access to sexual health services and information.

A limitation of using the results of the 2018 GPS as our dataset is that the survey contains few sexual health questions. Some aspects of sexual health are not covered in detail on the survey because data are already collected in other global surveys, as is the case with abortion. However, there remain gaps in data for certain aspects of sexual health, for example, services for transgender healthcare. These gaps call for additional research on how laws ensure access to services not covered by the 2018 GPS.

Finally, it is important to acknowledge that this analysis is limited to what is included in laws. The extent to which specific provisions in laws enable or hinder access to sexual health services and information is also dependent on the content of relevant policies, the broader legal framework of the country, and the degree of implementation of such laws and policies.

Despite these limitations, the content of the laws in this analysis provides important and novel insights into the types of laws ensuring access to sexual health services and information, the specific services or information ensured or prohibited, and characteristics that are explicitly protected in non-discrimination provisions. These results may guide further investigation to understand the relationship between such legal provisions, the implementation of these laws within countries, and relevant sexual health outcomes.

## Conclusion and way forward

This analysis provides novel insight into the nature of laws that govern access to sexual health information and services. Findings revealed variation in the types and numbers of laws uploaded, which and how many services are covered, and the extent to which non-discrimination is addressed. From the perspective of enhancing laws to be more supportive, the scattered nature and complexity in ascertaining where one should look for laws relevant to sexual health poses an inherent challenge. Large scale and more in-depth research on this topic, building on the methods and results of this analysis, would expand the evidence base on which future work could be carried out. Findings might usefully inform national legal and policy dialogues around sexual health to identify opportunities for positive change.

Looking ahead toward the post-2030 agenda, it is critical that the global community continue to take steps that further advance the realisation of the highest attainable standard of sexual health. Understanding the substantial variation in how laws ensure access to sexual health services and information, the laws’ strengths and weaknesses and their accountability mechanisms, is necessary for informing future advocacy, guidance, and the measurement of progress at country and global levels. Strengthening these legal provisions and their implementation is an essential element of the SDGs and for realisation of the right to health for everyone, everywhere.

## Provenance statement

This article was not commissioned and went through external peer review.
